# Development and evaluation of an ontology for non-invasive respiratory support in acute care

**DOI:** 10.1371/journal.pone.0348199

**Published:** 2026-05-04

**Authors:** Md Fantacher Islam, Jarrod Mosier, Vignesh Subbian

**Affiliations:** 1 College of Engineering, The University of Arizona, Tucson, Arizona, United States of America; 2 College of Medicine, The University of Arizona, Tucson, Arizona, United States of America; Centre Hospitalier Universitaire Farhat Hached de Sousse, TUNISIA

## Abstract

Managing patients with respiratory failure increasingly involves non-invasive respiratory support (NIRS) strategies to support respiration, often preventing the need for invasive mechanical ventilation. However, despite the rapidly expanding use of NIRS, there remains a significant challenge to its optimal use across all medical circumstances. It lacks a unified ontological structure, complicating guidance on NIRS modalities across healthcare systems. In this study, we introduced NIRS ontology to support knowledge representation in acute care settings by providing a unified framework that enhances data clarity and interoperability, laying the groundwork for future clinical decision-making research. We developed NIRS ontology using the Web Ontology Language (OWL) and Protégé to organize clinical concepts and relationships. To enable rule-based clinical reasoning beyond hierarchical structures, we added Semantic Web Rule Language (SWRL) rules. We evaluated logical reasoning using a sample of 6 patient scenarios and used SPARQL queries to retrieve and test targeted inferences. The ontology has 145 classes, 11 object properties, and 18 data properties across 949 axioms that establish concept relationships. To standardize clinical concepts, we added 392 annotations, including descriptive definitions based on controlled vocabularies. SPARQL queries successfully validated all test cases (rules) by retrieving appropriate patients’ outcomes: for instance, a patient treated with HFNC (high-flow nasal cannula) for 2 hours due to acute respiratory failure may avoid endotracheal intubation. This NIRS ontology formally represents domain-specific concepts, including ventilation modalities, patient characteristics, therapy parameters, and outcomes. SPARQL query evaluations across clinical scenarios confirmed the ontology’s ability to support rule‑based reasoning and therapy recommendations, providing a foundation for consistent documentation practices, integration into clinical data models, and advanced analysis of NIRS outcomes. In conclusion, this proof-of-concept NIRS ontology demonstrates how clinical concepts can be formally represented and queried, offering a foundation for future validation, EHR integration, and evidence-based refinement of NIRS practices.

## Background

Noninvasive respiratory support (NIRS) modalities have emerged as effective strategies for managing patients with acute respiratory failure, such as acute exacerbations of chronic obstructive pulmonary disease, cardiogenic pulmonary edema, and acute hypoxemic respiratory failure (AHRF) [1–3]. These strategies have expanded available respiratory support options beyond conventional oxygen or invasive mechanical ventilation [4]. Despite their clinical effectiveness, NIRS modalities such as Continuous Positive Airway Pressure (CPAP) and High-Flow Nasal Cannula (HFNC) originated in different contexts (CPAP for sleep medicine [5] and HFNC for neonatal care [6]) before being adopted in adult acute care settings [7]. Historically, these modalities were studied and documented separately, but the COVID-19 pandemic unified them into NIRS interventions for the massive influx of hypoxemic patients [8]. Consequently, data captured within Electronic Health Records (EHRs) tends to be fragmented, with each modality documented using distinct parameter sets, creating interoperability issues [9]. Nonetheless, the lack of standardized representations regarding the nature and use of NIRS too often limits reproducible cohort definitions, phenotyping, and cross-site comparability across the spectrum of heterogeneous respiratory failure conditions [10].

More specifically, a key challenge for clinician-scientists, research informaticians, and administrators is the lack of standardized methods for describing and categorizing NIRS modalities and their combinations, which complicates collecting and analyzing large-scale NIRS data [11]. This highlights challenges in using secondary EHR data, which are often heterogeneous and lack explicit domain knowledge, making interpretation difficult due to inconsistency, inaccuracy, and incompleteness [12]. These varying documentation practices within and between healthcare systems limit the ability to further real-world use and evidence related to the effectiveness and safety of NIRS [13]. These multifaceted challenges highlight the need for a domain-specific ontology and knowledge representation framework for NIRS to facilitate accurate information exchange and inform clinical decision-making across healthcare settings [14]. Recent studies have developed taxonomies to clarify complex terminologies, establish consistent vocabularies, and support more informed comparisons related to invasive mechanical ventilation [15,16]. Similarly, while domain-specific ontologies have been developed to standardize intensive care diagnoses, these frameworks do not capture the granular therapeutic parameters required for NIRS phenotyping [17]. Yet, a unified standardization approach specific to NIRS is missing.

While standard taxonomies provide essential lists of terms, they lack the semantic reasoning required to handle imperfect or conflicting data. Addressing these gaps through ontology-based approaches offers a promising solution for enhancing interoperability, enabling more advanced clinical queries, and providing a structured foundation for improving NIRS practices [18]. Ontologies provide formal, machine-interpretable representations that unify clinical data by explicitly defining domain-specific concepts and relationships, supporting semantic clarity and enabling meaningful data integration [14]. Although prior ontology efforts have addressed mechanical ventilation and temporal relationships in clinical events, such as the Time Event Ontology (TEO), which uses Allen’s interval algebra, a comprehensive ontology tailored to NIRS has yet to be developed [19].

The goal of this study is to develop a domain-specific ontology for Non-Invasive Respiratory Support (NIRS). Specifically, we aim to: [1] formalize the hierarchical relationships between distinct NIRS modalities, devices, and their parameters, [2] map these concepts to standard controlled vocabularies (e.g., SNOMED-CT, ICD-10, and MedDRA) to ensure interoperability, and [3] demonstrate the ontology’s applicability in clinical phenotyping.

## Methods

**Fig 1:**
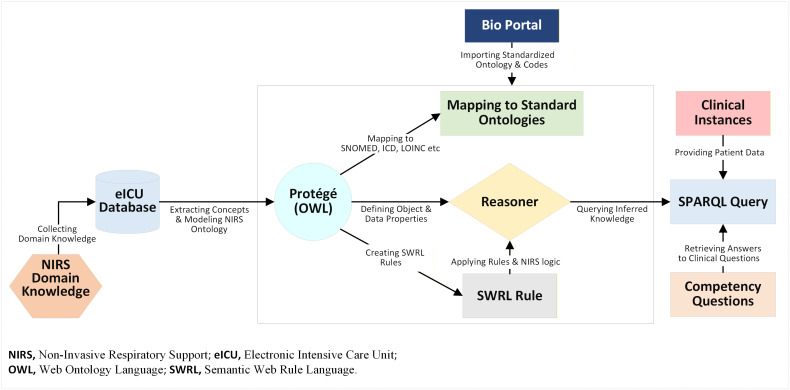
Workflow of the NIRS Ontology Development and Application.

**Fig 1** illustrates the end-to-end workflow, beginning with incorporating domain knowledge and extracting data elements from the eICU (Electronic Intensive Care Unit) collaborative research database, continuing through modeling in Protégé, and concluding with creating SWRL (Semantic Web Rule Language) rules and SPARQL queries to support competency-based insights [20].

### Data source

A part of this study involved the analysis of a publicly available eICU database v2.0, containing de-identified electronic health record data from over 200,000 admissions across 335 units at 208 United States Hospitals between 2014 and 2015 [20]. This study is exempt from institutional review board (IRB) approval due to the retrospective design, lack of direct patient intervention, and the security schema, for which the re-identification risk was certified as meeting safe harbor standards by an independent privacy expert (Privacert, Cambridge, MA) (Health Insurance Portability and Accountability Act Certification no. 1031219-2).

### Data extraction and clinical concepts for NIRS

We extracted clinical concepts and data on conditions commonly treated with NIRS modalities from five core tables in the eICU database: *respiratoryCharting*, *carePlanGeneral*, *diagnosis*, *nurseCharting*, and *treatment*. These tables provided key factors, including NIRS indications, modality, and settings. A review of published research informed our inclusion criteria, which aimed to cover all acute illnesses (e.g., sepsis, septic shock, pneumonia) [21,22] and decompensated chronic illnesses (e.g., cardiogenic pulmonary edema, COPD (chronic obstructive pulmonary disease), and OHS (Obesity Hypoventilation Syndrome)) typically managed with NIRS [23,24]. We also extracted data known to be either comorbidities or confounders associated with improved or worsened outcomes with NIRS [25–27]. Specific threshold values for data extraction (e.g., BMI cutoffs) and reasoning rules were selected based on clinical practice guidelines (such as the American Thoracic Society (ATS)) and peer-reviewed articles to ensure clinical relevance. To ensure accuracy, an intensivist reviewed and validated the underlying domain knowledge, including class hierarchies and safety thresholds to ensure alignment with real-world acute care workflows.

### Ontology construction and mapping

We constructed the NIRS ontology using Web Ontology Language (OWL) semantics and Protégé software to organize clinical concepts and relationships (**Fig 1**). We defined core superclasses and subclasses for NIRS strategies by reviewing published literature, collaborating with an intensivist through a formal iterative process, and analyzing the eICU database. This approach ensured that the ontology is intuitive for human users and interoperable with computational tools, including Protégé. To enhance semantic interoperability, we added annotations to leaf or terminal classes. For defining the concepts, we use ‘rdfs:label’ for readable names (e.g., “Chronic Obstructive Pulmonary Disease” for COPD), ‘rdfs:comment’ for a concise clinical definition, and ‘rdfs:seeAlso’ to link to resources such as BioPortal. To map concepts to standard vocabularies, we created a custom annotation ‘hasOntoCode,’ which holds vocabulary codes with IDs, such as SNOMED-CT: 13645005. A team of two informaticians performed these mappings through a two-round iterative process. The initial mapping was conducted using the BioPortal tool. We defined object and data properties to interrelate classes or subclasses and store associated values. Object properties establish relationships between classes (e.g., linking a patient’s indication to a therapy), while data properties capture parameters such as oxygen flow rate or FiO₂, with domains and ranges set for validation.

### Reasoning and inference in the NIRS ontology

After developing the NIRS hierarchy and annotations, we tested the logical reasoning using hypothetical patient records (instances or individuals) that represented real-world clinical scenarios. For instance, we would assign a patient with acute respiratory distress as HFNC (high-flow nasal cannula), with parameters such as flow rate and FiO₂, or noninvasive positive pressure ventilation (NIPPV), with IPAP (inspiratory positive airway pressure) and EPAP (expiratory positive airway pressure). We used the Pellet reasoner for checking consistency and inferring relationships and class memberships using OWL axioms. SWRL rules added domain-specific logic, enhancing clinical reasoning beyond basic hierarchies. These rules, created in Protégé, enabled complex decision-making, such as identifying critical conditions or suggesting therapies. SPARQL queries retrieved targeted data, allowing the analysis of trends such as therapy selection or outcomes. Together, these components formed a robust ontological framework for representing clinically meaningful knowledge, enabling semantic reasoning tasks such as classification and inference, and supporting integration of heterogeneous data sources for clinical decision-making and analysis.

### Ontology framework for NIRS

The NIRS ontology provides a structured representation of clinical knowledge related to non-invasive respiratory support, capturing the conditions that indicate its use, the specific modalities and parameters of therapy administered, and the observed patient responses or outcomes.

### Structural design of the ontology

**Fig 2:**
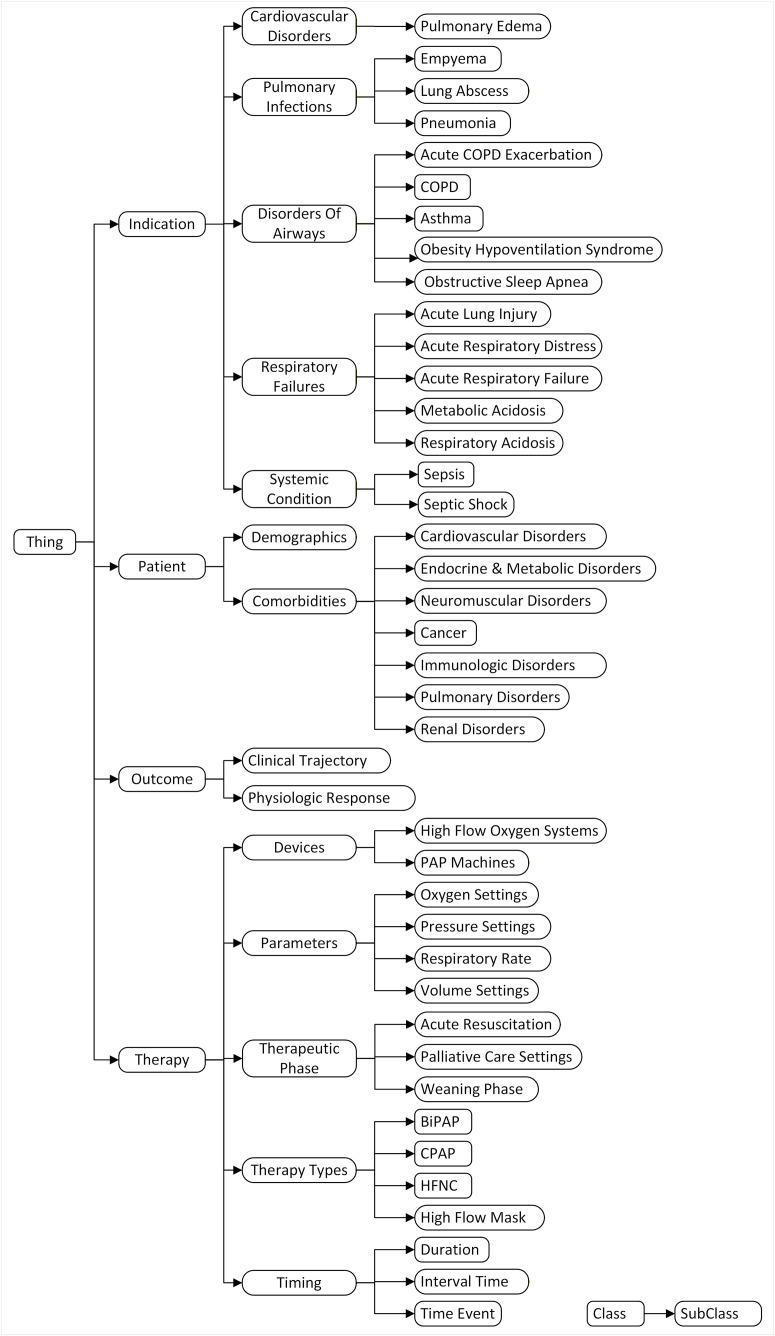
Class Hierarchy of the NIRS Ontology. BiPAP, Bilevel Positive Airway Pressure; CPAP, Continuous Positive Airway Pressure; COPD, Chronic Obstructive Pulmonary Disease; HFNC, High-Flow Nasal Cannula. Note: This figure displays only top-level classes. The hierarchical links represent semantic relationships and do not imply a deterministic clinical treatment pathway or causal relationship.

The NIRS ontology’s hierarchy begins with ‘Thing’ as the top-level class (**Fig 2**), serving as the universal root from which we derive primary superclasses: *Therapy*, *Indication*, *Patient*, and *Outcome*. The ‘Therapy’ superclass includes the required therapy details classes, such as the ‘TherapeuticPhase’ subclass, which captures the clinical intent of therapies, as defined by the ATS clinical practice guidelines [28–30]. This superclass also has other subclasses of device types, parameters, and timing, encompassing interventions, such as HFNC systems, positive airway pressure variants, and therapy duration. The ‘Patient’ superclass includes demographics, comorbidities, and other key patient characteristics (see Interactive Class Hierarchy).

Similarly, the ‘Outcome’ superclass represents clinical endpoints of NIRS strategies, such as avoiding intubation or requiring escalation due to insufficient initial treatment. This hierarchical structure, with superclasses branching into detailed subclasses, provides a robust framework for modeling diverse respiratory care scenarios.

### Object and data properties and conceptual links

Table 1 shows the key object properties defined within the NIRS ontology, indicating the relationships between domain and range classes. For example, the ‘*hasComorbidity*’ property links patients with their comorbidities, ‘*hasDevice*’ links therapies to their devices, and similarly, patients or therapies to specific outcomes are linked by the ‘*hasOutcome*’ property. Each property is associated with specific domains and ranges. These object properties ensure that classes logically interact and accurately represent clinical relationships within respiratory support scenarios.

**Table 1:** NIRS Ontology Object Properties.

Object Property	Domain	Range
hasComorbidity	Patient	Comorbidity
hasOutcome	Patient, TherapyType	Outcome
hasDevice	TherapyType	Device
hasTherapyType	Patient	TherapyType
hasRiskFactor	Patient	Comorbidity, Indication
hasParameter	TherapyType, Device	Parameter
isUsedIn	Device, Parameter	TherapyType
recommendedTherapyType	Patient	TherapyType
hasIndication	Patient	Indication
hasTiming	TherapyType	Timing
hasTreatmentEffect	Patient, TherapyType	Outcome

NIRS ontology uses data properties to capture numeric or time-related details that are essential to clinical decision-making. These properties store attributes such as a patient’s age using the data property name ‘*hasAge*’, body mass index using ‘*hasBMI*’, and ventilation settings using ‘*hasFiO2Value*’, ‘*hasPEEPValue*’, etc. Recording these details as data properties allows for logical reasoning in Protégé. Table 2 shows the data property definitions, indicating their domain, such as for Patient or PEEP, and the associated data type, such as integer or decimal. This definition enables ontology to enforce logical restrictions on how data is stored and how it would be queried.

**Table 2:** NIRS Ontology Data Properties.

Data Property	Domain	DataType
hasAge	Age	Integer
hasBMI	BMI	Float
hasDuration	Duration	Float
hasSFRatio	Patient	Float
hasPFRatio	Patient	Float
hasEPAPValue	EPAP	Float
hasFiO2Value	FiO_2_	Float
hasIPAPValue	IPAP	Float
hasOxygenFlowRate	OxygenFlowRate	Float
hasPEEPValue	PEEP	Float
hasTime	Timing	DateTime
hasRespiratoryRate	RespiratoryRate	Integer

BMI, Body Mass Index; EPAP, Expiratory Positive Airway Pressure; FiO₂, Fraction of Inspired Oxygen; IPAP, Inspiratory Positive Airway Pressure; PEEP, Positive End-Expiratory Pressure.

**Fig 3:**
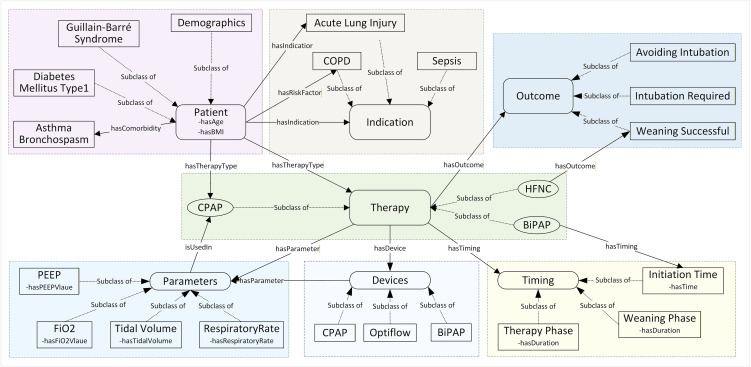
Conceptual Model of the NIRS Ontology with Class Hierarchies and Property Relationships. BiPAP, Bilevel Positive Airway Pressure; CPAP, Continuous Positive Airway Pressure; COPD, Chronic Obstructive Pulmonary Disease; FiO₂, Fraction of Inspired Oxygen; HFNC, High-Flow Nasal Cannula; PEEP, Positive End-Expiratory Pressure. Note: The arrows indicate that a relationship can exist between concepts (e.g., a Patient record containing both an Indication and a Therapy), and do not imply a deterministic clinical treatment pathway or causal relationship. Clinical decision logic and safety thresholds are handled separately by the SWRL rules described in Table 3.

**Fig 3** highlights the logical flow and relationships among ontology super classes. For instance, for a patient, the logical flow begins with the ‘Patient’ superclass, linking to clinical indications such as COPD through properties such as ‘*hasIndication*’ or connecting to comorbidities such as Asthma or Bronchospasm via ‘*hasComorbidity*’. In addition, ‘Patient’ superclass captures individual attributes using data properties such as ‘*hasAge*’ and ‘*hasBMI*’. The ‘Therapy’ superclass has therapeutic methods such as CPAP (continuous positive airway pressure) or devices such as BiPAP (bi-level positive airway pressure), identified by properties ‘*hasTherapyType*’ and ‘*hasDevice*’.

**Table 3:** SWRL Rule for Competency Questionnaire and Inferred Outcome.

Competency Question	Clinical Instances	SWRL Rule	Inferred Outcome/Class
Which clinical indications lead to the initiation of NIRS, and what measurable criteria define when a patient meets those indications?	If a Mild ARDS has a 200 ≤ P/F ratio ≤ 300 and PEEP ≥ 5, CPAP is recommended.	See rules in supporting information S1.1(Q1Rule 1)	Therapy Recommendation: CPAP
	If a COPD patient requires FiO₂ ≥ 0.40, BiPAP is recommended.	See rules in supporting information S1.1(Q1Rule 3)	Therapy Recommendation: BiPAP
How do the timing and duration of NIRS modalities influence patient outcomes?	If HFNC started within 3 hours, acute respiratory failure reduces intubation risk.	See rules in supporting information S1.1(Q3Rule 1)	Avoiding Intubation
	Weaning success likelihood increases when EPAP is reduced to 4 cmH2O, requiring a mean duration of 35 hours with BiPAP.	See rules in supporting information S1.1(Q3Rule 3)	Weaning Successful

ARDS, Acute Respiratory Distress Syndrome; BiPAP, Bilevel Positive Airway Pressure; COPD, Chronic Obstructive Pulmonary Disease; FiO₂, Fraction of Inspired Oxygen; HFNC, High-Flow Nasal Cannula.

The ‘Therapy’ class further specifies parameters such as PEEP using ‘*hasParameter*’ and stores their values using data properties such as ‘*hasPEEPValue*’ or ‘*hasFiO2Value*’, and it captures timing details, such as ‘Initiation Time’ through ‘*hasTiming*’. Finally, the logical sequence culminates in the ‘Outcome’ class, which defines results of therapy such as “Weaning Successful” or “Intubation Required” via ‘*hasOutcome*’. This structured flow ensures that the ontology accurately reflects realistic clinical scenarios encountered in NIRS.

### Clinical instances

We created clinical instances that are representative of practical scenarios from acute care settings by extracting examples of patient-ICU-stay from the eICU database to facilitate a precise evaluation of the NIRS ontology. Each scenario encompasses patient diagnoses, therapeutic interventions, clinical parameters, and outcomes, demonstrating the ontology’s practical usability and analytic capability. A few example scenarios are illustrated in Table 4, where the Time Offset column captures the timing of clinical events in minutes relative to unit admission and the NIRS modalities or device assertions during the ICU stay.

**Table 4:** Example of Clinical Instances for Patients.

Patient	Time Offset	Property Assertion	Class Instance
Patient_01	−11	hasComorbidity	Type 2 Diabetes
	12	hasIndication	COPD Exacerbation
	35	hasDeviceType	BiPAP/CPAP
	36	hasPEEPValue	5 cmH₂O
	36	hasFiO2Value	60%
Patient_02	113	hasIndication	Acute Respiratory Failure
	1060	hasFiO2Value	55%
	1060	hasPaO2Value	60 mm-Hg
	1060	haspHValue	7.47
	1090	hasFiO2Value	55%
	1190	hasRespiratoryRate	25 bpm
	1250	hasSpO2Value	93%
	1250 - 2870	hasDeviceType	HFNC
Patient_03	19945	hasRespiratoryRate	23 bpm
	20005	hasSpO2Value	96%
	20033	hasIndication	Pulmonary Edema
	20033	hasTherapyType	CPAP
Patient_04	1572	hasIndication	ARDS
	4212	hasFiO2Value	40%
	4212	hasPaO2Value	80 mm-Hg
	4270	hasPEEPValue	5 cmH₂O
	6090	hasTherapyType	CPAP
Patient_05	85585	hasIndication	Pneumonia
	85585	hasIndication	COPD Exacerbation
	85920	hasPEEPValue	14 cmH₂O
	85950	hasFiO2Value	100%
	86010	hasFiO2Value	100%
	86030	hasFiO2Value	40%
	86036	hasPEEPValue	15 cmH₂O
	86065	hasOutcome	Discharged and expired or Dead/Weaning Failure
Patient_06	354	hasPEEPValue	12 cmH₂O
	445	hasPaO2Value	247
	445	hasFiO2Value	100%
	598	hasIndication	Sepsis
	598	hasTherapyType	CPAP
	846	hasFiO2Value	60%

ARDS, Acute Respiratory Distress Syndrome; COPD, Chronic Obstructive Pulmonary Disease; BiPAP, Bilevel Positive Airway Pressure; CPAP, Continuous Positive Airway Pressure; HFNC, High-Flow Nasal Cannula.

For example, Patient_01 has an indication of COPD Exacerbation with a comorbidity of Type 2 Diabetes and is treated with BiPAP/CPAP with a PEEP of 5 cmH₂O and an FiO₂ of 60%. Separately, Patient_02 has an indication of Acute Respiratory Failure and is treated with HFNC with an FiO₂ of 55%, a respiratory rate of 25 bpm, and an SpO₂ of 93%. Similarly, Patient_04 has an indication of ARDS and is managed with CPAP, at a PEEP of 5 cmH₂O, with a P/F ratio of 200 mmHg (FiO₂ 40% and PaO₂ 80 mm-Hg, respectively).

### SWRL rules and clinical inferences

SWRL rules were integrated into the ontology to enable automated clinical reasoning. They link clinical criteria to clinical instances for clearly defined competency questions and their answers [27,31]. For example, according to the Berlin Definition of ARDS (acute respiratory distress syndrome), one SWRL rule in the NIRS ontology infers NIRS modalities (such as CPAP) as the recommended therapy for Mild ARDS patients with a P/F ratio between 200 and 300 mm-Hg and requiring a minimum PEEP of (≥5 cmH₂O) [32]. While another rule, according to the Global Initiative for Chronic Obstructive Lung Disease (GOLD) 2024 guidelines, suggests that COPD patients with a FiO₂ > 40% should receive BiPAP as the first-line treatment for conditions like respiratory acidosis or cardiogenic pulmonary edema to reduce the risk of intubation and mortality [33]**.** Similarly, according to the ATS Clinical Practice Guideline, patients with OHS require CPAP as the first-line therapy [34]. A selection of these competency questions and their corresponding rules is shown in Table 3. All other SWRL rules are described in the **S1 Appendix**.

Defining these rules in SWRL allows the ontology to dynamically recommend therapies or identify patients at greater risk of adverse outcomes. For instance, initiating HFNC within three hours of detecting acute hypoxemic respiratory failure can help avert intubation [35].

On the other hand, prolonged BiPAP use beyond 24–48 hours in patients with elevated pressure support and PEEP (10–12 cmH_2_O) may heighten the risk of intubation [33]. This integration of rule-based logic demonstrates how ontology supports critical clinical judgments and fosters data-driven insights in respiratory care.

### Ontology integration with standardized biomedical terminologies

To enhance semantic alignment with established standards, the NIRS ontology incorporates structured mappings to standardized biomedical coding systems. Using BioPortal’s search and cross-referencing tools, relevant classes were mapped to external vocabularies, such as ICD-10-CM, MedDRA, and SNOMED-CT. These mappings were performed by using annotations in Protégé for the leaf or terminal classes. A few examples of mappings, where each entry includes the class name, a descriptive label, an ontology code, an ontology link, and a brief comment highlighting its clinical use, are shown in **Table 5**. For instance, CPAP is mapped to HCPCS code E0601, referencing its role in maintaining constant airway pressure in non-invasive ventilation. BiPAP corresponds to a MedDRA code describing its dual-level pressure support. HFNC and High Flow Mask are linked to SNOMED-CT and MedDRA codes, respectively, both representing their function in delivering heated, humidified oxygen at high flow rates. These mappings strengthen interoperability, allowing ontology to integrate more effectively into clinical and research information systems.

**Table 5:** Mappings for NIRS therapy Type Classes.

Class	Label	Ontology Code	Ontology Link	Comment
CPAP	Continuous Positive Airway Pressure	HCPCS: E0601	HCPCS	A type of non-invasive respiratory support (NIRS) used to maintain constant pressure in the airways throughout the respiratory cycle
BiPAP	Bilevel Positive Airway Pressure	MEDDRA: 10064530	MedDRA	An NIRS ventilation mode delivering two pressure levels: a constant expiratory pressure (EPAP) and an increased pressure support for inspiration (IPAP).
HFNC	High Flow Nasal Cannula	SNOMED-CT: 426854004	SNOMED- CT	A non-invasive respiratory support that delivers heated, humidified oxygen at high flow rates (up to 60 L/min) via nasal prongs.
HighFlowMask	High Flow Mask Oxygen Therapy	MEDDRA: 10084914	MedDRA	A non-invasive respiratory support using heated, humidified oxygen at high flow rates (up to 15 L/min) to improve oxygenation.

## Results

The NIRS ontology consists of 145 classes (*Therapy*: 50, *Indication*: 25, *Patient*: 58, *Outcome*: 8), 11 object properties, 18 data properties, and 26 individuals (including 6 patient clinical scenarios). It was built upon 181 subclass axioms, supported by defined object property domains and ranges, as well as annotation properties such as ‘*rdfs:label’*, ‘*rdfs:comment’*, and external ontology codes. In total, 949 axioms were defined to capture semantic relationships among therapy parameters, clinical indications, comorbidities, timing, and outcomes.

**Fig 4:**
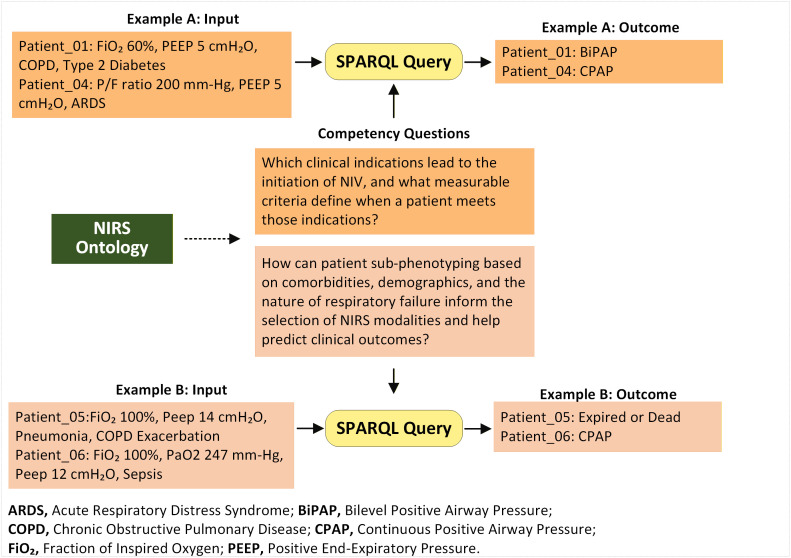
Evaluation of SWRL rule logic for competency questionnaire. (‘Example A or B: Input’) Patient’s scenario classes related to Competency question 1 or 2. (Example A or B: Outcome’) Patient’s scenario outcome classes retrieved by SPARQL.

To evaluate the reasoning ability to answer NIRS ontology’s competency questions, we applied SWRL rules to a sample of 6 patients extracted from the eICU database. SPARQL queries retrieved inferred answers to those questions and extracted instances where patients met specific, measurable criteria (**Fig 4**). For example, a patient (Patient_01) with a COPD indication and an FiO₂ requirement of 60% inferred a recommendation for the NIRS modality of BiPAP. Similarly, a patient (Patient_04) with Mild ARDS, a P/F ratio of 200 mm-Hg, and a PEEP value of 5 cmH₂O, was inferred to require CPAP (see rules in supporting information S1.1 in S1 Appendix).

Regarding risk level, a sepsis patient (Patient_06) with an FiO₂ value of 100% inferred a higher risk of intubation, while a patient (Patient_05) with COPD exacerbation requiring high FiO₂ of 100% with a P/F ratio of 247 mm-Hg (with a PaO_2_ value of 247 mm-Hg) inferred an elevated mortality risk due to weaning failure (see rules in supporting information S1.2 in S1 Appendix).

**Fig 5:**
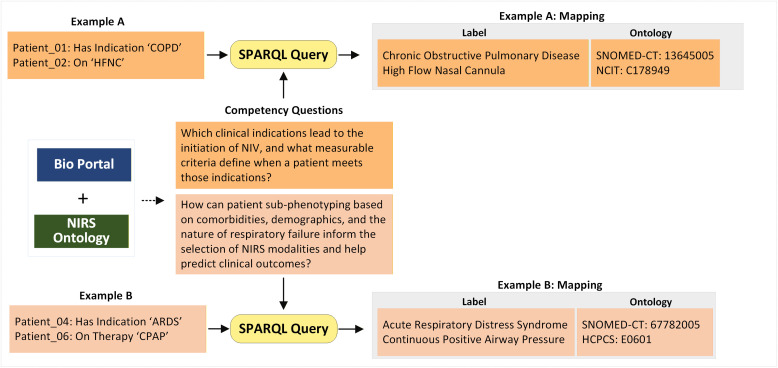
Retrieving Mapping Annotations by SPARQL. (Example **A)** Patient’s indications and outcomes classes related to Competency question 1. (Example **B)** Patient’s indications and therapy type classes related to Competency question 2. (Example A or B: Mapping) Shows retrieved classes, labels, or definitions and Ontology mapping.

We also evaluated the ontology’s capacity to support semantic interoperability. Out of 100 leaf or terminal classes, 87 were successfully mapped to standardized codes, a list of these mappings is provided in **S1 Table**. The remaining 13 classes, such as Oximizer, Optiflow, and Avoiding Intubation, represent domain-specific concepts that currently lack direct equivalents in standard terminologies. With the addition of annotations, the extracted outputs can include standardized vocabulary with codes and the descriptive definitions (‘rdfs:comment’) or full form (‘rdfs:label’) of different clinical concepts, depending on user needs. For instance, a patient (Patient_03) was recommended for BiPAP therapy with a COPD indication. In this case, the query may concern the details of COPD indications or BiPAP therapy, where COPD is annotated with the SNOMED-CT vocabulary using a concept code of 13645005, and BiPAP is annotated with MedDRA using a concept code of 10064530. Similarly, for a patient (Patient_06), who was at risk for intubation, the query can retrieve information about the risk of intubation or parameters with relevant vocabulary and concept codes (**Fig 5**).

## Discussion

In this study, we developed the NIRS ontology to provide a unified framework for NIRS modalities and introduced a semantic reasoning layer. Existing ontologies, such as the Time Event Ontology [18], and other standardized ICU diagnoses [16], lack therapeutic parameters needed for phenotyping NIRS interventions. While terminologies such as SNOMED CT and LOINC are essential for clinical documentation, they do not incorporate therapeutic reasoning or support machine-based inference across diverse data sources. Our framework addresses these limitations by linking clinical concepts with rule-based inference. For example, whereas standard queries rely on explicit coding to identify “Respiratory Failure”, the NIRS ontology uses SWRL rules to infer this physiological data (e.g., P/F ratio) and therapy settings.

The ontology design supports interoperability through both its structured class hierarchy and the use of annotation properties, such as ‘rdfs:label’, ‘rdfs:comment’, and ‘hasOntoCode’. The classes were organized to reflect core clinical concepts such as indications, therapy types, outcomes, and device settings, making them compatible with standardized vocabularies. We mapped approximately 87% of leaf classes to external terminologies, while domain-specific concepts without direct equivalents (e.g., proprietary device names) were defined using internal annotations. Annotations were applied selectively based on the relevance of each class in each scenario, allowing targeted mapping without unnecessary duplication. For example, the outcome “Intubation Required” was mapped to a concept in the NCI Thesaurus, while the therapy type BiPAP was linked to a MedDRA term. Mapping these terminal nodes to standard codes gives the NIRS ontology support for integration with diverse EHR settings.

The ontology demonstrates the inference capabilities of NIRS ontology for retrospective use, such as using SPARQL queries to identify cohorts with specific phenotypic features. While the existing classes and rules support therapy recommendations and outcomes, the scope of the NIRS ontology is limited to acute and decompensated chronic conditions. Furthermore, the current implementation features a set of 17 SWRL rules, a subset of which was validated against clinical instances to demonstrate technical feasibility. Additional rules are needed to support future questions involving more granular conditions or evolving therapy states. Expanding the ontology to include broader SWRL coverage would enhance its capacity to represent clinical complexity. This work is an early-stage effort, showing semantic reasoning over retrospective data, not readiness for clinical decision support. However, a prospective evaluation of ontology is required to validate its effectiveness across different EHR systems.

### Limitation

Our study has several limitations. First, the definition of the SWRL rule relies on guideline-based clinical scenarios, with expert review by an intensivist. However, the ontology has not been tested with retrospective analysis to measure its predictive accuracy. Second, although our ontology captures basic temporal concepts (e.g., duration), it does not fully represent complex, continuous time-series trends such as rapid desaturation over minutes, which are vital for real-time monitoring. Third, the thresholds in our rules (e.g., FiO₂ ≥ 0.40 for COPD) are derived from specific guidelines listed in the **S1 Appendix** and may not align with local protocols or clinical gray zones. These thresholds are flexible and can be updated as new information becomes available. Fourth, the ontology was internally validated using eICU data from 2014–2015. While the clinical concepts are still relevant, new modalities or interfaces developed after 2015 may require updates to the class hierarchy.

## Conclusion

This study provides a unified NIRS ontology that captures key components, including patient characteristics, clinical indications, therapy parameters, and outcomes. The ontology was developed using real-world eICU data and encoded with OWL semantics. It allows rule-based reasoning and patient-specific query execution through SWRL and SPARQL. The ontology facilitates a clear representation of therapy logic across various clinical contexts by incorporating NIRS modalities in a consistent, machine-readable structure. This NIRS framework demonstrates how complex, context-dependent therapies can be formally represented and queried, offering a foundation for more consistent documentation practices, integration into clinical data models, and advanced analysis of NIRS outcomes. However, this work represents a foundational, proof-of-concept framework that requires prospective validation, independent cohort evaluation, and EHR integration testing before any clinical implementation.

## Supporting information

S1 Appendix SWRL Rules and SPARQL Queries. This file contains the complete definitions of the Semantic Web Rule Language (SWRL) rules used for clinical reasoning, along with the corresponding SPARQL queries developed to retrieve and validate ontology inferences.
(DOCX)

S1 Table Mapping of Leaf or Terminal Nodes in NIRS Ontology. (DOCX)
