# Supplementary material for: Development and evaluation of an ontology for non-invasive respiratory support in acute care
Source: PLoS One. 2026 May 4;21(5):e0348199. doi: 10.1371/journal.pone.0348199 (PMC13138654; doi:10.1371/journal.pone.0348199)
Supplement: S1 Appendix — This file contains the complete definitions of the Semantic Web Rule Language (SWRL) rules used for clinical reasoning, along with the corresponding SPARQL queries developed to retrieve and validate ontology inferences. (DOCX) [file pone.0348199.s001.docx]

# **S1 APPENDIX**

S1.1 SWRL Rules

This section describes the Semantic Web Rule Language (SWRL) rules that are designed to answer specific competency questions by enabling inference over the ontology.

S1.1.1 Competency Question (Q1):

*Which clinical indications lead to the initiation of noninvasive ventilation and what measurable criteria when a patient meets those indications?*

**Q1Rule 1:**

Rule Name: MildARDS_ModeratePFRatio_CPAP

Rule Guideline: 2012 Journal of the American Medical Association’s Berlin Definition Report for ARDS (1)

Rule Comment: If a Mild ARDS has 200 ≤ P/F ratio ≤ 300 and PEEP/CPAP ≥ 5, CPAP is recommended.

Rule Code:

Patient(?pt) ^ hasIndication(?pt, AcuteRespiratoryDistress) ^ hasPFRatio(?pt, ?pfr) ^ swrlb:lessThanOrEqual(?pfr, 300) ^ swrlb:greaterThanOrEqual(?pfr, 200) ^ hasPEEPValue(?pt, ?peep) ^ swrlb:greaterThanOrEqual(?peep, 5) -> recommendedTherapyType(?pt, CPAP)

**Q1Rule 2:**

Rule Name: NIRS_For_CHF_Respiratory_Distress

Rule Guideline: 2021 European Society of Cardiology Guidelines Report (2)

Rule Comment: For CHF patients with respiratory distress (RR >25 breaths/min, SpO2 <90%), NIRS modality is recommended to alleviate dyspnea and reduce the rate of mechanical endotracheal intubation.

Rule Code:

Patient(?pt) ^ hasIndication(?pt, CongestiveHeartFailure) ^ hasRespiratoryRate(?pt, ?rr) ^ swrlb:greaterThan(?rr, 25) ^ hasSpO2Value(?pt, ?spo2) ^ swrlb:lessThan(?spo2, 90) -> recommendedTherapyType(?pt, NIRSModality)

**Q1Rule 3:**

Rule Name: COPD_HighFiO2_BiPAP

Rule Guideline: 2025 Global Initiative for Chronic Obstructive Lung Disease Report

Rule Comment: If a COPD patient requires FiO₂ ≥ 0.40, BiPAP is recommended.

Rule Code:

Patient(?pt) ^ hasIndication(?pt, COPD) ^ hasFiO2Value(?pt, ?fiO2) ^ swrlb:greaterThanOrEqual(?fiO2, 0.40) -> recommendedTherapyType(?pt, BiPAP)

**Q1Rule 4:**

Rule Name: HighIPAP_HighPEEP_Intubation

Rule Guideline: 2016 British Thoracic Society and Intensive Care Society Report (3)

Rule Comment: If high IPAP (>20–30 cmH₂O) or EPAP (>10–12 cmH₂O) are required without improvement in pH, PaCO₂, or respiratory rate despite optimization, the patient is at high risk of NIRS failure and needing intubation.

Rule Code:

Patient(?pt) ^ hasIPAPValue(?pt, ?ipap) ^ swrlb:greaterThan(?ipap, 20) ^ hasEPAPValue(?pt, ?epap) ^ swrlb:greaterThan(?epap, 10) -> hasOutcome(?pt, IntubationRequired)

**Q1Rule 5:**

Rule Name: LowFiO2_LowPEEP_WeaningSuccess.

Rule Guideline: 2008 National Institutes of Health Network Mechanical Ventilation Protocol Summary

Rule Comment: Patients stable on FiO₂ ≤0.40 and PEEP ≤8 cm H₂O are candidates for spontaneous breathing trials, indicating potential weaning success if sustained post-extubation.

Rule Code:

Patient(?pt) ^ hasFiO2Value(?pt, ?fiO2) ^ swrlb:lessThanOrEqual(?fiO2, 0.40) ^ hasPEEPValue(?pt, ?peep) ^ swrlb:lessThanOrEqual(?peep, 8) -> hasOutcome(?pt, WeaningSuccessful)

**Q1Rule 6:**

Rule Name: NIRS_Failure_Leads_To_Intubation

Rule Guideline: 2021 Pulmonology Journal Non-invasive Respiratory Support Algorithm Report (4)

Rule Comment: If P/F Ratio < 100 or PaCO2 increases by 20% from basal levels, the patient is at risk of Non-invasive respiratory support failure and needs intubation.

Rule Code:

Patient(?pt) ^ hasPFRatio(?pt, ?pfr) ^ swrlb:lessThan(?pfr, 100) -> hasOutcome(?pt, IntubationRequired)

**Q1Rule 7:**

Rule Name: ObesityHypoventilationSyndrome_BiPAP

Rule Guideline: 2012 New South Wales Agency and 2010 German Pneumology Society Guidelines (5)

Rule Comment: For OHS patients with hypercapnia (PaCO2 >45 mmHg), recommend BiPAP if CPAP fails to resolve hypoventilation.

Rule Code:

Patient(?pt) ^ hasIndication(?pt, ObesityHypoventilationSyndrome) ^ hasPaCO2(?pt, ?paco2) ^ swrlb:greaterThan(?paco2, 45) ^ hasTherapyType(?pt, CPAP) -> recommendedTherapyType(?pt, BiPAP)

**Q1Rule 8:**

Rule Name: Pneumonia_Hypoxemia_HFNC (6)

Rule Guideline: 2024 Annals of Intensive Care Narrative Review

Rule Comment: For Pneumonia patients with hypoxemia (SpO2 <90% on >10 L/min standard O2) and high work of breathing (RR >25), recommend HFNC.

Rule Code:

Patient(?pt) ^ hasIndication(?pt, Pneumonia) ^ hasSpO2(?pt, ?spo2) ^ swrlb:lessThan(?spo2, 90) ^ hasOxygenFlowRate(?pt, ?flow) ^ swrlb:greaterThan(?flow, 10) ^ hasRespiratoryRate(?pt, ?rr) ^ swrlb:greaterThan(?rr, 25) -> recommendedTherapyType(?pt, HFNC)

S1.1.2 Competency Question (Q2):

How can patient sub-phenotyping based on comorbidities, demographics, and the nature of respiratory failure inform the selection of NIRS modalities and help predict clinical outcomes?

**Q2Rule 1:**

Rule Name: Elderly_COVID_NIRS_Failure

Rule Guideline: 2023 Internal and Emergency Medicine Report (7)

Rule Comment: Elderly (≥ 75 years) COVID-19 patients have a higher risk of NIRS failure or mortality.

Rule Code:

Patient(?pt) ^ hasIndication(?pt, COVID19) ^ hasAge(?pt, ?age) ^ swrlb:greaterThanOrEqual(?age, 75) -> hasOutcome(?pt, WeaningFailure)

**Q2Rule 2:**

Rule Name: Neuromuscular_NIRS_Modality

Rule Guideline: 2016 British Thoracic Society and Intensive Care Society Guideline Report (3)

Rule Comment: If a neuromuscular disease is diagnosed, the patient is recommended to the NIRS Modality.

Rule Code:

Patient(?pt) ^ hasIndication(?pt, NeuromuscularDisorders) ->

recommendedTherapyType(?pt, NIRSModality)

**Q2Rule 3:**

Rule Name: OHS_CPAP_FirstLine_Therapy

Rule Guideline: 2019 Official American Thoracic Society Clinical Practice Guideline (8)

Rule Comment: For Obesity Hypoventilation patients require CPAP as the first line therapy.

Rule Code:

Patient(?pt) ^ hasIndication(?pt, ObesityHypoventilationSyndrome) -> recommendedTherapyType(?pt, CPAP)

**Q2Rule 4:**

Rule Name: Sepsis_NIV_Survival_Threshold

Rule Guideline: 2025 BioMed Central Pulmonary Medicine Report (9)

Rule Comment: Sepsis patients with PaO2/FiO2 ≥ 241 show increased survival rates and avoid intubation with initial NIRS modality.

Rule Code:

Patient(?pt) ^ hasIndication(?pt, Sepsis) ^ hasPFRatio(?pt, ?pfr) ^ swrlb:greaterThanOrEqual(?pfr, 241) ^ hasTherapyType(?pt, ?t) ^ TherapyType(?t) -> hasOutcome(?pt, AvoidingIntubation)

**Q2Rule 5:**

Rule Name: COPD_Oxygen_Target_Avoids_Acidosis

Rule Guideline: 2017 British Thoracic Society Emergency Oxygen Use Guideline (10)

Rule Comment: COPD exacerbations requiring high FiO₂ (>0.60) elevate mortality risk through worsened hypercapnia and acidosis.

Rule Code:

Patient(?pt) ^ hasIndication(?pt, COPD) ^ hasFiO2Value(?pt, ?fio2) ^ swrlb:greaterThan(?fio2, 0.60) -> hasOutcome(?pt, WeaningFailure)

S1.1.3 Competency Question (Q3):

How do the timing and duration of NIRS modalities influence patient outcomes?

**Q3Rule 1:**

Rule Name Early_HFNC_Reduces_Intubation

Rule Guideline: 2015 New England Journal of Medicine High Flow Oxygen Report (11)

Rule Comment: If HFNC is started within 3 hours, it may reduce intubation risk in patients with P/F ≤ 200 mm Hg.

Rule Code:

Patient(?pt) ^ hasPFRatio(?pt, ?pfr) ^ swrlb:lessThanOrEqual(?pfr, 200) ^ hasTherapyType(?pt, HFNC) ^ hasTiming(?pt, ?time) ^ swrlb:lessThanOrEqual(?time, 3) -> hasOutcome(?pt, AvoidingIntubation)

**Q3Rule 2:**

Rule Name: Prolonged_BiPAP_Leads_To_Intubation

Rule Guideline: 2022 Scientific Reports Deep Learning Model (12)

Rule Comment: Intubation after BiPAP duration > 24 hours is associated with fewer Ventilator Free Days.

Rule Code:

Patient(?pt) ^ hasTherapyType(?pt, BiPAP) ^ hasDuration(?pt, ?duration) ^ swrlb:greaterThan(?duration, 24) -> hasOutcome(?pt, IntubationRequired)

**Q3Rule 3:**

Rule Name: BiPAP_Weaning_EPAP_Target

Rule Guideline: 2009 Indian Journal of Critical Care Medicine Report (13)

Rule Comment: Weaning success likelihood increases when EPAP is reduced to 4 cmH2O, typically requiring a mean duration of 35 hours with BiPAP.

Rule Code:

Patient(?pt) ^ hasTherapyType(?pt, BiPAP) ^ hasEPAPValue(?pt, ?epap) ^ swrlb:lessThanOrEqual(?epap, 4) ^ hasDuration(?pt, ?duration) ^ swrlb:greaterThanOrEqual(?duration, 35) -> hasOutcome(?pt, WeaningSuccessful)

S1.1.4 Competency Question (Q4):

How do key therapy parameters (e.g., IPAP, EPAP, FiO₂) change in response to a patient’s evolving respiratory status?

**Q4Rule 1:**

Rule Name: OHS_EPAP_for_Hypoxemia

Rule Guideline: 2016 British Thoracic Society Guideline Report (3)

Rule Comment: For Obesity Hypoventilation Syndrome patients, titrating EPAP to 10-15 cmH₂O recruits collapsed lungs and improves oxygenation.

Rule Code:

Patient(?pt) ^ hasIndication(?pt, ObesityHypoventilationSyndrome) ^ hasEPAPValue(?pt, ?epap)^ swrlb:lessThanOrEqual(?ipap, 10) ^ swrlb:greaterThan(?ipap, 15)

-> hasOutcome(?pt, ImprovementOxygenation)

**Q4Rule 2:**

Rule Name: Low_SF_Ratio_Predicts_Failure

Rule Guideline: 2022 Scientific Reports Pediatric Bi-level Positive Airway Pressure Failure Study (12)

Rule Comment: For patients with an S/F ratio < 264, the likelihood of intubation increases.

Rule Code:

Patient(?pt) ^ hasSFRatio(?pt, ?sfr) ^ swrlb:lessThan(?sfr, 264) -> hasOutcome(?pt, IntubationRequired)

**Q4Rule 3:**

Rule Name: Increase_IPAP_for_Hypercapnia

Rule Guideline: 2016 British Thoracic Society Guideline Report (14)

Rule Comment: Higher IPAP (typically 20-30 cmH₂O) improves PaCO₂ clearance and reduces the need for intubation in hypercapnic COPD patients.

Rule Code:

Patient(?pt) ^ hasIndication(?pt, COPD) ^ hasIPAPValue(?pt, ?ipap)^ swrlb:lessThanOrEqual(?ipap, 20) ^ swrlb:greaterThan(?ipap, 30) -> hasOutcome(?pt, AvoidingIntubation)

S1.2 SPARQL Query

This section presents SPARQL queries to retrieve and validate information from the ontology in response to the competency questions.

S1.2.1 Query for Q1 Rule 1

PREFIX nirs: <http://www.semanticweb.org/fantacher/ontologies/2025/1/nirs#>

SELECT

?patient

(STR(?pfr) AS ?PFRatio_Value)

(STR(?peep) AS ?PEEP_Value)

(?indication AS ?Indication_Type)

(?therapy AS ?Recommended_Therapy)

WHERE {

?patient a nirs:Patient .

?patient nirs:hasIndication nirs:AcuteRespiratoryDistress .

# Match the rule's specific data points

?patient nirs:hasPFRatio ?pfr .

?patient nirs:hasPEEPValue ?peep .

OPTIONAL { ?patient nirs:hasIndication ?indication . }

OPTIONAL { ?patient nirs:recommendedTherapyType ?therapy . }

# Filter based on Q1Rule1 logic: 200 <= PFR <= 300 AND PEEP >= 5

FILTER (?pfr >= 200 && ?pfr <= 300 && ?peep >= 5)

}

S1.2.2 Query for Q1 Rule 3

PREFIX nirs: <http://www.semanticweb.org/fantacher/ontologies/2025/1/nirs#>

SELECT ?patient

(?indication AS ?Indication_Type)

(STR(?fio2) AS ?FiO2_Value)

(?therapy AS ?Recommended_Therapy)

WHERE {

?patient a nirs:Patient .

?patient nirs:hasIndication nirs:COPD .

OPTIONAL { ?patient nirs:hasIndication ?indication . }

OPTIONAL { ?patient nirs:hasFiO2Value ?fio2. }

OPTIONAL { ?patient nirs:recommendedTherapyType ?therapy . }

FILTER(?fio2 >= 0.40)

}

S1.2.3 Query for Q2 Rule 4

PREFIX nirs: <http://www.semanticweb.org/fantacher/ontologies/2025/1/nirs#>

SELECT

?patient

(STR(?pfr) AS ?PFRatio_Value)

(?currentTherapy AS ?Current_Therapy)

(?indication AS ?Indication_Type)

(?outcome AS ?Predicted_Outcome)

WHERE {

?patient a nirs:Patient .

?patient nirs:hasIndication nirs:Sepsis .

?patient nirs:hasPFRatio ?pfr .

?patient nirs:hasTherapyType ?currentTherapy .

# UPDATED LOGIC MATCHING SWRL: TherapyType(?t)

# This checks that the therapy is an instance of the TherapyType class

# (Reasoning must be enabled for this to catch subclasses like CPAP/BiPAP)

?currentTherapy a nirs:TherapyType .

OPTIONAL { ?patient nirs:hasIndication ?indication . }

OPTIONAL { ?patient nirs:hasOutcome ?outcome . }

# Filter based on Q2Rule4 logic: Only PFR >= 241 is needed here

FILTER (?pfr >= 241)

}

S1.2.4 Query for Q2 Rule 5

PREFIX nirs: <http://www.semanticweb.org/fantacher/ontologies/2025/1/nirs#>

SELECT

?patient

(STR(?fio2) AS ?FiO2_Value)

(?indication AS ?Indication_Type)

(?outcome AS ?Predicted_Outcome)

WHERE {

?patient a nirs:Patient .

?patient nirs:hasIndication nirs:COPD .

?patient nirs:hasFiO2Value ?fio2 .

OPTIONAL { ?patient nirs:hasIndication ?indication . }

OPTIONAL { ?patient nirs:hasOutcome ?outcome . }

# Filter based on Q2Rule5 logic: FiO2 > 0.60

FILTER (?fio2 > 0.60)

}

S1.3 SPARQL Query with RDFS Mapping Annotations

This section presents SPARQL queries designed to retrieve and verify annotations assigned to ontology classes.

S1.3.1 Query with Annotations for Q1 Rule 1

PREFIX rdfs: <http://www.w3.org/2000/01/rdf-schema#>

PREFIX nirs: <http://www.semanticweb.org/fantacher/ontologies/2025/1/nirs#>

SELECT

?patient

(STR(?pfr) AS ?PFRatio_Value)

(STR(?peep) AS ?PEEP_Value)

(?therapy AS ?Recommended_Therapy)

(?indication AS ?Indication_Type)

(STR(?label) AS ?Label)

(STR(?ontologyCode) AS ?OntologyCode)

(STR(?comment) AS ?Comment)

WHERE {

?patient a nirs:Patient .

?patient nirs:hasIndication nirs:AcuteRespiratoryDistress .

# Match the rule's specific data points

?patient nirs:hasPFRatio ?pfr .

?patient nirs:hasPEEPValue ?peep .

OPTIONAL { ?patient nirs:hasIndication ?indication . }

OPTIONAL { ?patient nirs:recommendedTherapyType ?therapy . }

OPTIONAL { ?indication rdfs:label ?label . }

OPTIONAL { ?indication nirs:hasOntoCode?ontologyCode . }

OPTIONAL { ?indication rdfs:comment ?comment . }

# Filter based on Q1Rule1 logic: 200 <= PFR <= 300 AND PEEP >= 5

FILTER (?pfr >= 200 && ?pfr <= 300 && ?peep >= 5)

}

S1.3.1 Query with Annotations for Q1 Rule 3

PREFIX rdfs: <[http://www.w3.org/2000/01/rdf-schema#](http://www.w3.org/2000/01/rdf-schema)>

PREFIX nirs: <[http://www.semanticweb.org/fantacher/ontologies/2025/1/nirs#](http://www.semanticweb.org/fantacher/ontologies/2025/1/nirse)>

SELECT ?patient

(STR(?fio2) AS ?FiO2_Value)

(?therapy AS ?Recommended_Therapy)

(?indication AS ?Indication_Type)

(STR(?label) AS ?Label)

(STR(?ontologyCode) AS ?OntologyCode)

(STR(?comment) AS ?Comment)

WHERE {

?patient a nirs:Patient .

?patient nirs:hasIndication nirs:COPD .

OPTIONAL { ?patient nirs:hasIndication ?indication . }

OPTIONAL { ?patient nirs:hasFiO2Value ?fio2. }

OPTIONAL { ?patient nirs:recommendedTherapyType ?therapy . }

OPTIONAL { ?indication rdfs:label ?label . }

OPTIONAL { ?indication nirs:hasOntoCode?ontologyCode . }

OPTIONAL { ?indication rdfs:comment ?comment . }

FILTER(?fio2 >= 0.40)

}

S1.3.3 Query with Annotations for Q2 Rule 4

PREFIX rdfs: <http://www.w3.org/2000/01/rdf-schema#>

PREFIX nirs: <http://www.semanticweb.org/fantacher/ontologies/2025/1/nirs#>

SELECT

?patient

(STR(?pfr) AS ?PFRatio_Value)

(?currentTherapy AS ?Current_Therapy)

(?outcome AS ?Predicted_Outcome)

(?indication AS ?Indication_Type)

(STR(?label) AS ?Label)

(STR(?ontologyCode) AS ?OntologyCode)

(STR(?comment) AS ?Comment)

WHERE {

?patient a nirs:Patient .

?patient nirs:hasIndication nirs:Sepsis .

?patient nirs:hasPFRatio ?pfr .

?patient nirs:hasTherapyType ?currentTherapy .

# UPDATED LOGIC MATCHING SWRL: TherapyType(?t)

# This checks that the therapy is an instance of the TherapyType class

# (Reasoning must be enabled for this to catch subclasses like CPAP/BiPAP)

?currentTherapy a nirs:TherapyType .

OPTIONAL { ?patient nirs:hasIndication ?indication . }

OPTIONAL { ?patient nirs:hasOutcome ?outcome . }

OPTIONAL { ?indication rdfs:label ?label . }

OPTIONAL { ?indication nirs:hasOntoCode?ontologyCode . }

OPTIONAL { ?indication rdfs:comment ?comment . }

# Filter based on Q2Rule4 logic: Only PFR >= 241 is needed here

FILTER (?pfr >= 241)

}

S1.3.4 Query with Annotations for Q2 Rule 5

PREFIX rdfs: <http://www.w3.org/2000/01/rdf-schema#>

PREFIX nirs: <http://www.semanticweb.org/fantacher/ontologies/2025/1/nirs#>

SELECT

?patient

(STR(?fio2) AS ?FiO2_Value)

(?outcome AS ?Predicted_Outcome)

(?indication AS ?Indication_Type)

(STR(?label) AS ?Label)

(STR(?ontologyCode) AS ?OntologyCode)

(STR(?comment) AS ?Comment)

WHERE {

?patient a nirs:Patient .

?patient nirs:hasIndication nirs:COPD .

?patient nirs:hasFiO2Value ?fio2 .

OPTIONAL { ?patient nirs:hasIndication ?indication . }

OPTIONAL { ?patient nirs:hasOutcome ?outcome . }

OPTIONAL { ?indication rdfs:label ?label . }

OPTIONAL { ?indication nirs:hasOntoCode?ontologyCode . }

OPTIONAL { ?indication rdfs:comment ?comment . }

# Filter based on Q2Rule5 logic: FiO2 > 0.60

FILTER (?fio2 > 0.60)

}

# **References**

1. Ranieri VM, Rubenfeld GD, Taylor Thompson B, Ferguson ND, Caldwell E, Fan E, Camporota L, Slutsky AS. Acute respiratory distress syndrome: the Berlin Definition. JAMA: Journal of the American Medical Association. 2012 Jun 20;307(23).

2. Falsetti L, Guerrieri E, Zaccone V, Santini S, Giovenali L, Pierdomenico G, et al. A Comparison of Different Guidelines for the Treatment of Acute Heart Failure and Their Extensibility to Emergency Departments: A Critical Reappraisal. Journal of Clinical Medicine. 2025 May 17;14(10):3522.

3. Davidson AC, Banham S, Elliott M, Kennedy D, Gelder C, Glossop A, et al. BTS/ICS guideline for the ventilatory management of acute hypercapnic respiratory failure in adults. Thorax. 2016 Apr;71(Suppl 2):ii1–35.

4. Winck JC, Scala R. Non-invasive respiratory support paths in hospitalized patients with COVID-19: proposal of an algorithm. Pulmonology. 2021 July 30;27(4):305–12.

5. Wang Z, Wilson M, Dobler CC, Morrow AS, Beuschel B, Alsawas M, Benkhadra R, Seisa M, Mittal A, Sanchez M, Daraz L. Noninvasive Positive Pressure Ventilation in the Home (2020).

6. Thille AW, Balen F, Carteaux G, Chouihed T, Frat JP, Girault C, et al. Oxygen therapy and noninvasive respiratory supports in acute hypoxemic respiratory failure: a narrative review. Annals of Intensive Care. 2024 Oct 18;14(1):158.

7. Crisafulli E, Sartori G, Vianello A, Maroccia A, Lepori E, Quici M, et al. Use of non-invasive respiratory supports in high-intensity internal medicine setting during the first two waves of the COVID-19 pandemic emergency in Italy: a multicenter, real-life experience. Internal and Emergency Medicine. 2023 Sep;18(6):1777-87.

8. Mokhlesi B, Masa JF, Brozek JL, Gurubhagavatula I, Murphy PB, Piper AJ, et al. Evaluation and Management of Obesity Hypoventilation Syndrome. An Official American Thoracic Society Clinical Practice Guideline. American journal of respiratory and critical care medicine. 2019 Aug 1;200(3):e6-24.

9. Lu Y, Zhang J, Zhang W, Shi H, Wang K, Li Z, et al. Impact of initial ventilation strategies on in-hospital mortality in sepsis patients: insights from the MIMIC-IV database. BMC Pulmonary Medicine. 2025 Apr 1;25(1):147.

10. O’Driscoll BR, Howard LS, Earis J, Mak V. British Thoracic Society Guideline for oxygen use in adults in healthcare and emergency settings. BMJ open respiratory research. 2017 May 15;4(1).

11. Frat JP, Thille AW, Mercat A, Girault C, Ragot S, Perbet S, et al. High-Flow Oxygen through Nasal Cannula in Acute Hypoxemic Respiratory Failure. New England Journal of Medicine. 2015 Jun 4;372(23):2185-96.

12. Im DD, Laksana E, Ledbetter DR, Aczon MD, Khemani RG, Wetzel RC. Development of a deep learning model that predicts Bi-level positive airway pressure failure. Scientific Reports. 2022 May 26;12(1):8907.

13. Prasad SBN, Khanna R. Role of noninvasive ventilation in weaning from mechanical ventilation in patients of chronic obstructive pulmonary disease: Indian Journal of Critical Care Medicine: Peer-reviewed, Official Publication of Indian Society of Critical Care Medicine. 2009 Oct;13(4):207.

14. Ghosh D, Elliott MW. Acute non-invasive ventilation – getting it right on the acute medical take. Clinical Medicine. 2019 May;19(3):237–42.
