# Supplementary material for: Development and evaluation of an ontology for non-invasive respiratory support in acute care
Source: PLoS One. 2026 May 4;21(5):e0348199. doi: 10.1371/journal.pone.0348199 (PMC13138654; doi:10.1371/journal.pone.0348199)
Supplement: S1 Table — (DOCX) [file pone.0348199.s002.docx]

# **S2 Table.** Mapping of NIRS Ontology Leaf or Terminal Nodes

| Superclass | Leaf/End Class (Concept) | Standard Vocabulary | Concept Code |
| --- | --- | --- | --- |
| Indication | Pulmonary Edema | SNOMEDCT | 19242006 |
|  | AECOPD | SNOMEDCT | 195951007 |
|  | Asthma | SNOMEDCT | 195967001 |
|  | COPD | SNOMEDCT | 13645005 |
|  | Obesity Hypoventilation Syndrome (OHS) | ICD9CM | 278.03 |
|  | Obstructive Sleep Apnea (OSA) | SNOMEDCT | 78275009 |
|  | COVID-19 | SNOMEDCT | 840539006 |
|  | Empyema | SNOMEDCT | 312682007 |
|  | Lung Abscess | SNOMEDCT | 73452002 |
|  | Pneumonia | SNOMEDCT | 233604007 |
|  | Acute Lung Injury | NCIT | C155766 |
|  | Acute Respiratory Distress Syndrome (ARDS) | SNOMEDCT | 67782005 |
|  | Hypercapnic Respiratory Failure | SNOMEDCT | 709109004 |
|  | Hypoxemic Respiratory Failure | SNOMEDCT | 10676831000119101 |
|  | Mixed Respiratory Failure | N/A | N/A |
|  | Metabolic Acidosis | SNOMEDCT | 59455009 |
|  | Respiratory Acidosis | SNOMEDCT | 12326000 |
|  | Sepsis | SNOMEDCT | 91302008 |
|  | Septic Shock | SNOMEDCT | 76571007 |
| Outcome | Avoiding Intubation | N/A | N/A |
|  | Intubation Required | NCIT Code | C178949 |
|  | Weaning Failure | MEDDRA | 10066829 |
|  | Successful Weaning | SNOMEDCT | 404998008 |
|  | Improvement Oxygenation | SNOMEDCT | 284032008 |
|  | Reduction Hypercapnia | SNOMEDCT | 29596007 |
| Patient | PaO2/FiO2 Ratio | IOBC | 201406062517222943 |
|  | SpO2/FiO2 Ratio | N/A | N/A |
|  | Hematologic Malignancy | DOID | 2531 |
|  | Lung Cancer | LNC | LA15687-9 |
|  | Metastatic Lung Cancer | LNC | LA28289-9 |
|  | Thoracic Cancer | DOID | 5093 |
|  | Body Mass Index (BMI) | LNC | 39156-5 |
|  | Smoking Status | SNOMEDCT | 308512009 |
|  | Age | N/A | N/A |
|  | Sex | N/A | N/A |
|  | Lung Cancer | LNC | LA15687-9 |
|  | Metastatic Lung Cancer | LNC | LA28289-9 |
|  | Thoracic Cancer | DOID | 5093 |
|  | Biventricular | ICD10CM | I50.82 |
|  | CHF Diastolic | ICD10CM | I50.3 |
|  | CHF Left Sided | ICD10CM | I50.1 |
|  | CHF Right Sided | ICD10CM | I50.81 |
|  | CHF Systolic | ICD10CM | I50.2 |
|  | Cor-Pulmonale | SNOMEDCT | 83291003 |
|  | Hypertension Heart Failure | SNOMEDCT | 84114007 |
|  | Valvular Regurgitation | SNOMEDCT | 10337008 |
|  | Valvular Stenosis | NCIT | C62433 |
|  | DiabetesMellitusType1 | ICD10CM | E10 |
|  | DiabetesMellitusType2 | ICD10CM | E11 |
|  | AIDS | SNOMEDCT | 62479008 |
|  | Immunosuppression | SNOMEDCT | 38013005 |
|  | Sarcoidosis | SNOMEDCT | 31541009 |
|  | SLE | SNOMEDCT | 55464009 |
|  | Vasculitis | SNOMEDCT | 31996006 |
|  | ALS | SNOMEDCT | 86044005 |
|  | Guillain-Barre Syndrome | ICD10 | G61.0 |
|  | Multiple Sclerosis | SNOMEDCT | 24700007 |
|  | Neuropathy | SNOMEDCT | 386033004 |
|  | Post-Polio Syndrome | MEDDRA | 10036239 |
|  | Chronic Alcoholic Myopathy | SNOMEDCT | 838376007 |
|  | Malignant Hyperthermia | SNOMEDCT | 405501007 |
|  | Muscular Dystrophy | SNOMEDCT | 73297009 |
|  | Myopathy Critical-Illness | SNOMEDCT | 443819006 |
|  | Rhabdomyolysis | SNOMEDCT | 240131006 |
|  | Asthma Bronchospasm | SNOMEDCT | 195967001 |
|  | Asthma Mild | SNOMEDCT | 370218001 |
|  | Pleural Effusion CHF | SNOMEDCT | 90727007 |
|  | Diabetes Insipidus | ICD10CM | E23.2 |
|  | Acute Kidney Injury | SNOMEDCT | 14669001 |
|  | Chronic Kidney Disease | SNOMEDCT | 709044004 |
| Therapy | HFNC | SNOMEDCT | 426854004 |
|  | Optiflow | N/A | N/A |
|  | Oximizer | N/A | N/A |
|  | Vapotherm | N/A | N/A |
|  | BiPAP | MEDDRA | 10064530 |
|  | CPAP | HCPCS | E0601 |
|  | PaCO2 | SNOMEDCT | 25284008 |
|  | pH | CMO | 0000379 |
|  | TotalCO2 | OCHV | C0860709 |
|  | FiO2 | LNC | LP286756-4 |
|  | Oxygen Flow Rate | SNOMEDCT | 42708100 |
|  | EPAP | SNOMEDCT | 699113009 |
|  | IPAP | RCTV2 | 8726.00 |
|  | PEEP | SNOMEDCT | 250854009 |
|  | Pressure Support | LNC | LP73175-9 |
|  | Set Respiratory Rate | SNOMEDCT | 86290005 |
|  | Spontaneous Respiratory Rate | SNOMEDCT | 271625008 |
|  | Inspiratory Flow Rate | LNC | LP101893-8 |
|  | Inspiratory Pressure Set | SNOMEDCT | 417071008 |
|  | Tidal Volume | LNC | LP188693-8 |
|  | Acute Resuscitation | SNOMEDCT | 439569004 |
|  | Ceiling Of Therapy | N/A | N/A |
|  | Do Not Intubate | N/A | N/A |
|  | Post-Extubation Support | SNOMEDCT | 718089001 |
|  | Weaning Support | N/A | N/A |
|  | High Flow Mask | MEDDRA | 10084914 |
|  | Cumulative Time | N/A | N/A |
|  | Therapy Duration | SNOMEDCT | 261773006 |
|  | Therapy Interval | SNOMEDCT | 261774000 |
|  | Weaning Interval | N/A | N/A |
|  | Initiation Time | LNC | MTHU046569 |
|  | Pause Time | SNOMEDCT | 250821007 |
|  | Termination Time | SNOMEDCT | 397898000 |
